# Experimental intranasal infection reveals broad tissue tropism of bovine coronavirus

**DOI:** 10.1186/s13567-025-01703-9

**Published:** 2026-01-03

**Authors:** Hyung-Chul Cho, Youngjun Kim, Jaehyeok Song, Seok-Jin Cho, Min-Ho Park, Jongho Kim, Jinho Park, Kyoung-Seong Choi

**Affiliations:** 1 Department of Animal Science and Biotechnology, College of Ecology and Environmental Science, Kyungpook National University, Sangju, 37224 Republic of Korea; 2 Department of Veterinary Internal Medicine, College of Veterinary Medicine, Jeonbuk National University, Iksan, 54596 Republic of Korea; 3 Animal Hospital, Hanwoo (Korean Indigenous Cattle) Genetic Improvement Center, National Agricultural Cooperative Federation, Seosan, 31948 Republic of Korea; 4 Department of Ecological Science, College of Ecology and Environmental Science, Kyungpook National University, Sangju, 37224 Republic of Korea; 5 Animal Disease Diagnostic Division, Animal and Plant Quarantine Agency, Gimcheon, 39660 Republic of Korea

**Keywords:** Bovine coronavirus, epithelial cells, histological lesions, tissue tropism, viral antigen

## Abstract

Bovine coronavirus (BCoV) causes neonatal calf diarrhea, winter dysentery, and respiratory disease worldwide. However, its primary replication site remains uncertain, and systemic studies on tissue tropism are limited. We, therefore, intranasally inoculated colostrum-fed Holstein calves (6–13-day-old) with a BCoV isolate derived from diarrheic calf, and monitored clinical signs, gross and histopathological lesions, antigen distribution, and viral replication in multiple tissues. All calves were seropositive before challenge. Infected animals developed diarrhea without respiratory manifestations. Viral RNA appeared first in nasal swabs and subsequently in feces, yet viral load in nasal secretions was not associated with clinical signs. Apart from enlargement of the mesenteric lymph nodes, no characteristic gross lesions were observed. Digital RT-PCR and immunohistochemistry detected BCoV in tonsil, trachea, lung, liver, kidney, abomasum, and both small and large intestines, indicating epithelial tropism at each site. Although the inoculum originated from a diarrheic calf, histopathology revealed enteritis, mild interstitial pneumonia, tonsillitis, and hepatitis. These data indicate that BCoV initiates replication in respiratory epithelia and subsequently disseminates to the digestive tract, with no correlation between gross pathology and viral detection. We conclude that BCoV exhibits broad tissue tropism and may contribute to disorders beyond classical respiratory and enteric disease, thereby refining the current understanding of BCoV pathogenesis.

**Supplementary Information:**

The online version contains supplementary material available at 10.1186/s13567-025-01703-9.

## Introduction

Bovine coronavirus (BCoV), a pneumoenteric *Betacoronavirus* 1, causes diarrhea and respiratory disease in cattle. Infection across age groups compromises weight gain and milk yield, generating substantial economic losses [1–4]. In the gastrointestinal tract, BCoV spreads from the duodenum to the large intestine, where villous atrophy and other mucosal lesions induce hemorrhagic–mucoid diarrhea [5–7]. Respiratory involvement includes interstitial pneumonia and epithelial damage of nasal turbinate, trachea, and lung [7–9]. Notably, BCoV is frequently detected in clinically healthy cattle [10–13].

BCoV belongs to the genus *Betacoronavirus*, which also comprises human coronavirus (OC43HCoV-OC43), severe acute respiratory syndrome-related coronavirus (SARS-CoV), Middle East respiratory syndrome coronavirus, and severe acute respiratory syndrome coronavirus 2 (SARS-CoV-2) [14]. In the most recent taxonomy, BCoV, HCoV-OC43, human enteric coronavirus HKU23, canine respiratory coronavirus, porcine hemagglutinating encephalomyelitis virus, equine coronavirus, and yak coronavirus have been grouped with the single species *Betacoronavirus* 1 [15]. Owing to their marked antigenic and genetic similarity, members of *Betacoronavirus* 1 are considered host-range variants of a common ancestral virus [16, 17]. Several coronaviruses exhibit broad tissue tropism and readily cross species barriers [4, 18]. BCoV operates as a quasispecies and can infect the small and large intestines, lungs, and occasionally the brain [19–22].

Historically regarded as a primary enteric pathogen with major economic consequences for the cattle industry [23], BCoV has more recently gained recognition as a contributor to bovine respiratory disease complex (BRDC) or “shipping fever” in feedlot cattle of various ages [1, 3, 24, 25]. BRDC causes considerable morbidity and mortality in 6–10-month-old beef cattle shortly after feedlot entry [26]; however, the precise role of BCoV in BRDC remains unresolved. The virus has been detected in respiratory samples from clinically healthy [27] and sick cattle [20, 28], and multiple studies implicate BCoV in respiratory illness [20, 22, 29–31].

BCoV spreads by both the fecal–oral and aerosol respiratory routes [32]. After entry, the virus replicates in epithelial cells of the respiratory and gastrointestinal tracts, resulting in shedding through nasal secretions and feces. Consequently, the mode of transmission dictates the initial replication sites and can influence subsequent shedding patterns [33]. Our recent experimental work showed that, irrespective of inoculation route, BCoV first replicates in the respiratory tract and subsequently in the gastrointestinal tract [34]. Consistent with this finding, BCoVs are shed in both feces and nasal secretions, regardless of the primary replication site [7, 11, 35]. Despite these advances, key aspects of BCoV pathogenesis—including the detailed transmission mechanism and the occurrence of viremia—remain incompletely understood [7, 8, 34].

Current evidence indicates that BCoV possesses dual respiratory and enteric tropism [34]. Viruses isolated from either site are antigenically indistinguishable [1], although suitable antigenic or genetic differences have occasionally been reported [36]. Nevertheless, disease outcome appears largely independent of the origin of the infecting strain [34, 37]. Owing to its large genome and rapid evolution, it remains uncertain whether enteric and respiratory BCoVs can be consistently distinguished antigenically [36, 38]. Recent analyses found no genetic or phylogenetic segregation between enteric and respiratory isolates but did identify clustering by sampling year [38, 39]. Moreover, isolates from either intestinal or respiratory origin replicate in both tissues when administered to gnotobiotic or colostrum-deprived calves [37]. Collectively, these observations suggest that a strict distinction between enteric and respiratory BCoV is no longer tenable.

We intranasally inoculated colostrum-fed Holstein calves with a BCoV isolate obtained from a diarrheic calf. Clinical signs, gross and histopathological lesions, viral antigen distribution, and viral replication across tissues were assessed to determine the primary replication site and tissue tropism.

## Materials and methods

### Ethics statement

This study was approved by the Institutional Animal Care and Use Committee at Kyungpook National University (No. KNU-2022–0110). All experimental procedures involving animals were conducted in strict accordance with relevant guidelines and regulations.

### Virus preparation, sequencing, and phylogenetic analyses

Fecal samples (200 mg) from a diarrheic BCoV-positive calf were processed for virus isolation as previously described [34]. After freeze–thaw cycles, supernatants were screened for BCoV by conventional RT-PCR and real-time RT-PCR targeting the spike (S) (920 bp) and the nucleocapsid (N) (87 bp) genes, respectively (Table 1). For sequencing analysis, PCR products amplified from the S gene were directly sequenced (Bioneer, Daejeon, ROK) and the obtained sequence (MW881214) was aligned using ClustalX (v.2.0) with reference strains. Phylogenetic trees were constructed on the basis of the TN93 + G + I model using MEGA 10 with 1000 bootstrap values. Virus propagation and titration were performed in Madin–Darby bovine kidney cells as previously described [40].

**Table 1:** **Primers used for bovine coronavirus detection in this study**

Method	Target	Primer	Sequences (5′–3′)	Amplicon size (bp)
Conventional RT-PCR	S	S1F	ATGTTTTTGATACTTTTAATTTCC	920
		S1R	ACACCAGTAGATGGTGCTAT	
Real-time RT-PCR	N	NF	CTAGTAACCAGGCTGATGTCAATACC	87
		NR	GGCGGAAACCTAGTCGGAATA	

S: spike, N: nucleocapsid.

### Animals

Prior to inoculation, blood and fecal samples were collected from all colostrum-fed Holstein calves (6–13 days old). Plasma was separated from blood, and nucleic acids were extracted from fecal suspensions using an AccuPrep Stool DNA extraction kit (Bioneer, Daejeon, ROK) and RNAiso Plus reagent (TaKaRa, Tokyo, Japan) according to the manufacturers’ instructions. *Cryptosporidium parvum* was screened using conventional PCR [41], whereas bovine viral diarrhea virus [42], group A bovine rotavirus (BoRVA) [43], and BCoV were assessed using real-time RT-PCR. All PCR results, including BCoV from nasal discharge, were negative; however, all calves were seropositive for BCoV by enzyme-linked immunosorbent assay (ELISA). All calves were clinically normal and exhibited no coughing, nasal discharge, or diarrhea. In each trial, four calves received 6 mL of intranasal BCoV (10^3.6^ TCID_50_/mL), and two calves served as controls. The animals were housed separately after inoculation. The experiment was repeated twice, yielding a total of 12 calves.

### Antibody measurement by ELISA

BCoV-specific antibodies were measured with a commercial indirect ELISA kit (SVANOVIR^®^ BCoV-Ab, Uppsala, Sweden) according to the manufacturer’s instructions. Plasma was added to 96-well plates precoated with BCoV antigen and incubated for 1 h at 37 °C. Optical density (OD) was read at 450 nm (Tecan, Männedorf, Switzerland). Percent positivity (PP) was calculated by dividing sample OD by the OD of the kit positive control. Calculated PP values greater than 10 were considered positive. The assay measures anti-BCoV IgG; neutralizing antibody titers were not determined.

### Samples collection

Infected calves were monitored daily, and respiratory signs plus feces color and consistency were scored (Table 2). Health status was assessed using the Calf Health Scoring Chart [44]. Only two clinical parameters—nasal discharge and feces—were evaluated. Nasal discharge was graded 0–3: 0, serous; 1, unilateral cloudy; 2, bilateral, excessive mucus; and 3, copious, bilateral mucopurulent nasal discharge. Fecal consistency was graded 0–3: 0, normal; 1, semi-formed (pasty); 2, loose; and 3, watery. Nasal and rectal swabs were collected at 1, 3, 5, 7, and 9 days post-infection (dpi) from inoculated and control calves. All calves were euthanized at 9 dpi. Anesthesia was induced with intramuscular administration of xylazine (Rompun, Elanco, USA; 0.03 mg/kg). After induction, euthanasia was performed by an intravenous administration of T-61 (MSD Animal Health, USA; 0.1 mL/kg). Tissue samples were subsequently collected and transported on ice. To assess other respiratory viral infections, lung samples were tested for bovine herpesvirus-1 [45], bovine respiratory syncytial virus, and bovine parainfluenza virus type 3 [46]; all results were negative.

**Table 2:** **Clinical signs in six Holstein calves experimentally infected with enteric BCoV**

Calf no	Colostrum intake	BCoV Ab	Onset of clinical signs, dpi (duration, days)	
			Diarrhea	Respiratory signs
1	+	+	3 dpi (6)	–
2	+	+	1 dpi (8)	–
3	+	+	3 dpi (6)	–
4	+	+	3 dpi (6)	–
5	+	+	1 dpi (5)	–
6	+	+	3 dpi (1)	–
7	+	+	5 dpi (1)	–
8	+	+	3 dpi (7)	–
C1	+	+	–	–
C2	+	+	–	–
C3	+	+	–	–
C4	+	+	–	–

All BCoV-infected calves passed loose diarrhea (score 2) and exhibited yellow diarrhea.

BCoV: bovine coronavirus, C: control calves, dpi: days post-infection, *–* no clinical signs.

### BCoV detection by real-time RT-PCR

All nasal and fecal samples were used for BCoV detection by N gene real-time RT-PCR. Viral RNA was extracted from nasal and rectal swabs using the AccuPrep^®^ Viral RNA Extraction Kit (Bioneer, Daejeon, ROK) according to the manufacturer’s instructions. Total RNA from tissue homogenates was extracted with the RNeasy Mini Kit (Qiagen, Hilden, Germany). Amplification was performed with the One Step TB Green^®^ PrimeScript™ RT-PCR Kit (Takara, Shiga, Japan). Ct values < 30 were considered positive. Each run included positive and negative controls.

### Detection of BCoV in tissue samples using RT-dPCR

RT-dPCR was carried out on the QIAcuity Digital PCR System (Qiagen, Hilden, Germany). Reactions (final volume 40 μL) contained 4× OneStep Advanced Probe Master Mix, 100× OneStep RT Mix, 400 nM of each primer, 200 nM of probe, RNA template, and PCR-grade water, and were loaded into 26 K 24-well Nanoplates (Qiagen). Reverse transcription proceeded at 50 °C for 40 min, followed by enzyme activation step at 95 °C for 2 min. The amplification program comprised 40 cycles of 95 °C for 5 s and 60 °C for 30 s, generating approximately 25 000 partitions per sample. Thresholds were defined in QIAcuity Software Suite v2.1.8 using internal positive and negative controls; the threshold was then manually refined against the positive control. Samples exceeding 0 copies/μL were deemed positive.

### Histopathology and IHC

Tissue samples were fixed immediately in 10% neutral-buffered formalin, shipped to IDEXX Laboratories (Westbrook, ME, USA), and processed for hematoxylin–eosin staining. Two blinded diagnostic pathologists evaluated the slides.

For immunohistochemistry (IHC), 5-μm paraffin sections were deparaffinized, rehydrated through graded alcohols, and subjected to heat-induced antigen retrieval in sodium citrate buffer (pH 6.0) for 30 min. After cooling to room temperature, sections were incubated with 3% H_2_O_2_ in methanol for 10 min to quench endogenous peroxidase activity. Nonspecific binding was blocked, and sections were incubated with a primary anti-BCoV antibody (clone BC6-4, NDSU Veterinary Diagnostic Laboratory, Fargo, ND, USA; 1:500). The following day, sections were incubated with the DISCOVERY universal secondary antibody (Ventana Medical Systems, Oro Valley, AZ, USA) for 1 h at room temperature, washed, and then incubated with the VECTASTAIN^®^ Elite ABC-HRP Kit (Vector Laboratories, Burlingame, CA, USA) for 30 min. Signals were visualized with the DAB Peroxidase Substrate Kit (Vector). Slides were rinsed, counterstained, mounted, examined by light microscopy, and photographed. Negative controls were incubated with isotype-matched IgG at the same dilution as the primary antibody.

## Results

### Phylogenetic analysis

**Figure 1:**
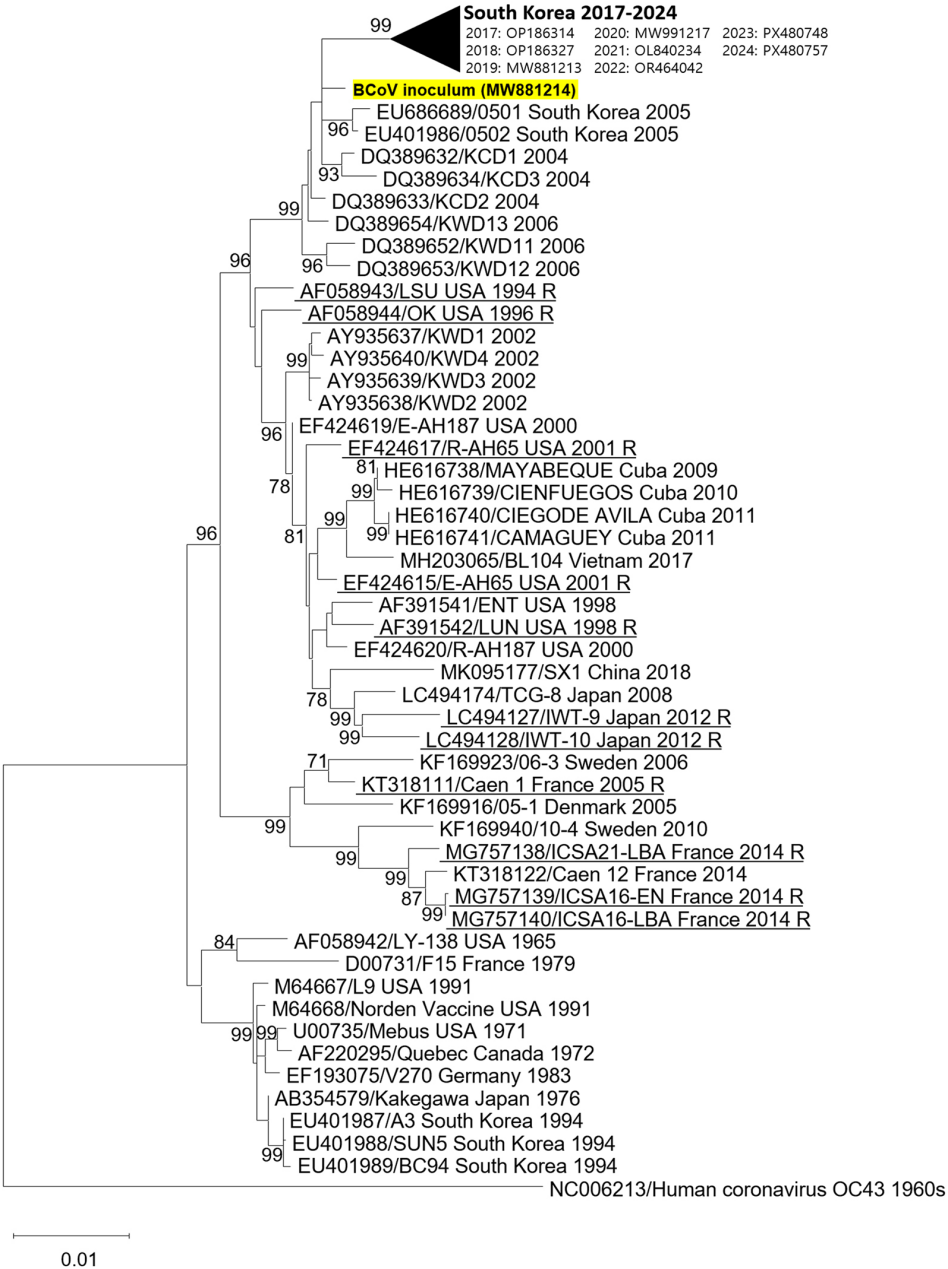
**Phylogenetic tree of bovine coronavirus spike gene sequences reported worldwide.** The tree was generated with the maximum-likelihood method under the TN93 + G + I substitution model, and bootstrap values (1000 replicates) > 70% are omitted. The inoculum sequence is highlighted with a yellow box. Respiratory BCoVs are underlined and indicated with R.

Phylogenetic analysis showed that the inoculum, isolated from a diarrheic calf (enteric BCoV), clustered with Korean enteric BCoVs reported since 2004, with 98.5–99.2% nucleotide identity but formed a distinct clade from the 2002 Korean enteric BCoVs (Figure 1). The isolate also shared 95.8–97.2% identity with respiratory BCoV strains reported in other countries. Notably, the analysis did not clearly separate enteric from respiratory BCoVs.

### Clinical observations

**Figure 2:**
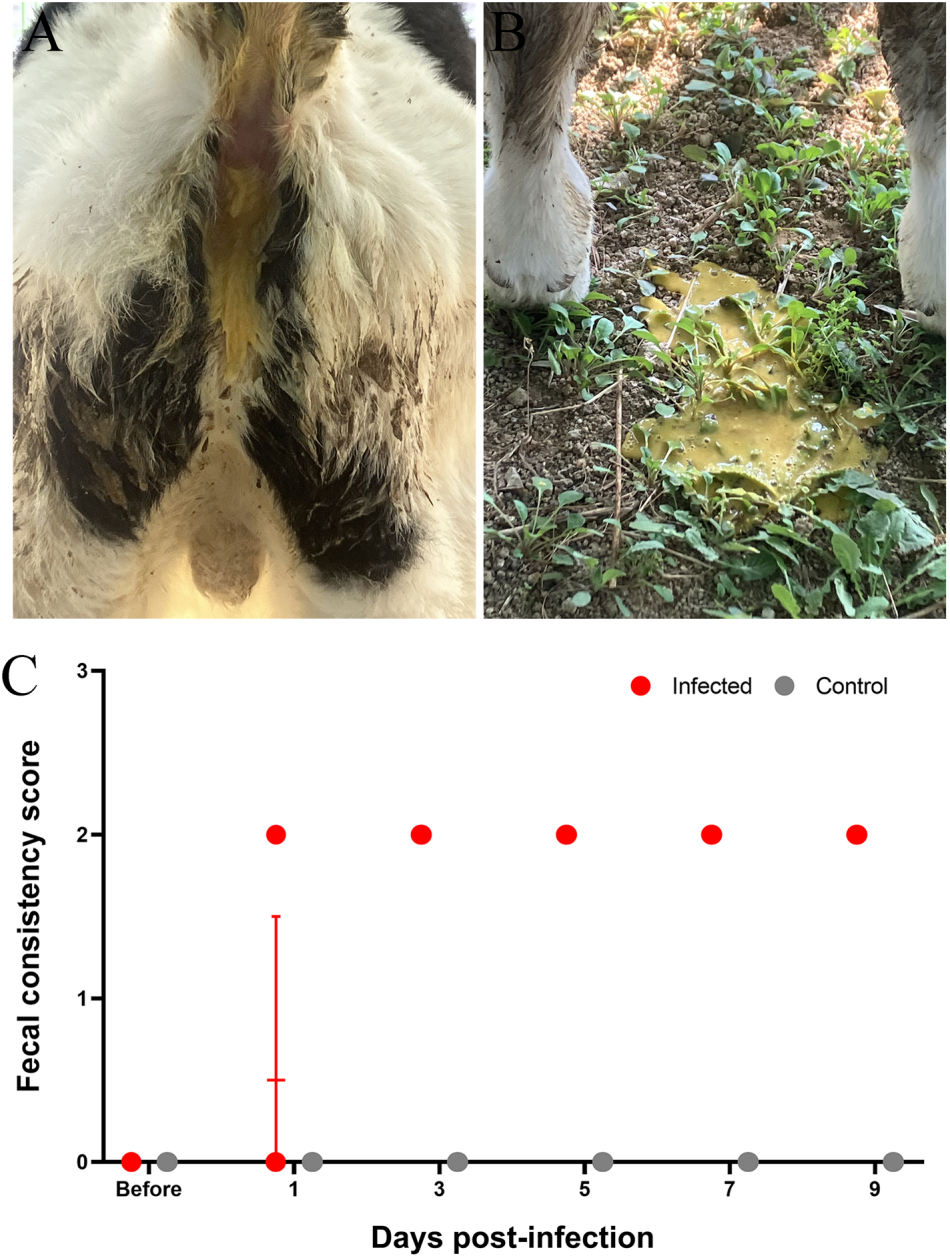
**Clinical manifestations in Holstein calves intranasally infected with enteric BCoV.** All infected calves developed watery diarrhea during the study (**A**) and showed yellowish diarrhea (**B**), whereas the control calves were normal (**C**). Diarrhea index was evaluated in the control and BCoV-infected groups (**D**).

All BCoV-infected Holstein calves developed diarrhea but showed no signs of anorexia, depression, nasal discharge, or coughing. Diarrhea began in two calves at 1 dpi, in five at 3 dpi, and in one at 5 dpi (Table 2). Diarrhea lasted 1–8 days. Feces were loose (Figure 2A) and yellow (Figure 2B) in every infected calf. Control calves remained clinically normal (Figure 2C). Infected calves were assigned a diarrhea score of 2 (Figure 2D).

### BCoV shedding

**Figure 3:**
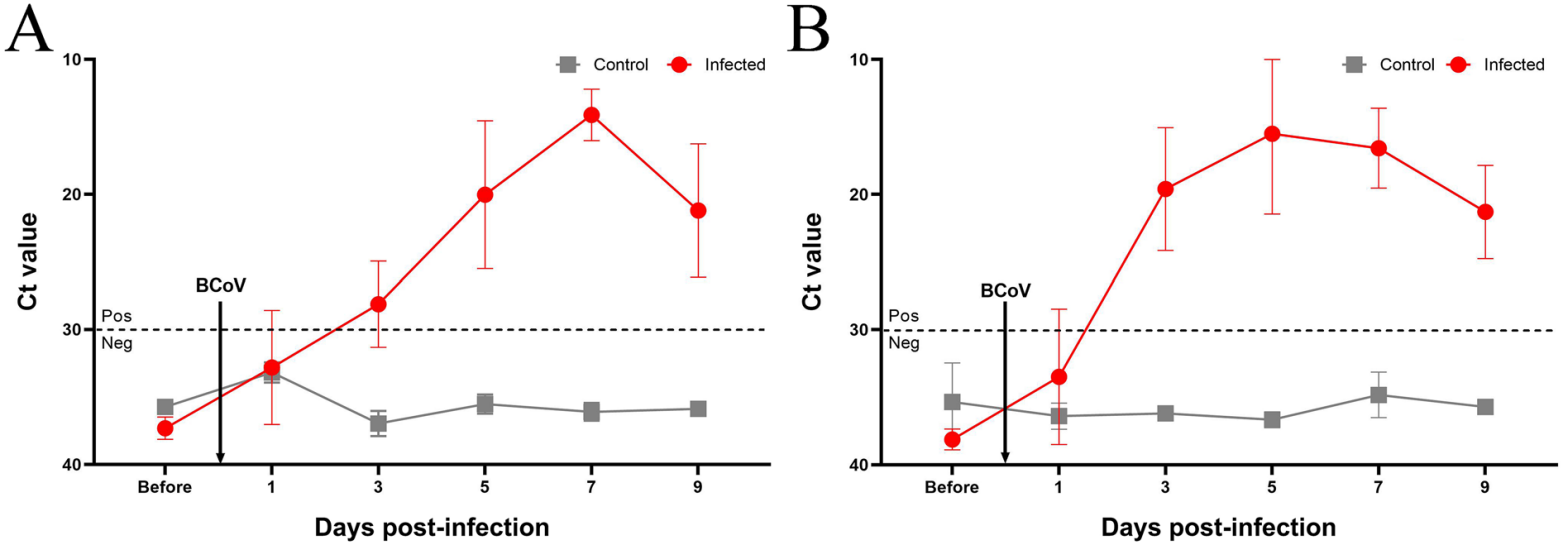
**BCoV shedding patterns from fecal (A) and nasal (B) swabs collected from intranasally infected calves.** Real-time RT-PCR targeting the nucleocapsid gene was performed; samples with cycle thresholds < 30 were considered positive. Viral RNA was detected in fecal and nasal swabs but was absent in control calves.

Virus detection in fecal and nasal swab samples was assessed by real-time RT-PCR. As shown in Figure 3A, BCoV in fecal samples was detectable in a single calf at 1 dpi and in two calves at 3 dpi. All infected calves shed viral RNA in their feces from 5 to 9 dpi, peaking at 7 dpi (Figure 3A). Viral RNA was detected in nasal swabs from two calves at 1 dpi; from 3 to 9 dpi, all infected calves exhibited nasal shedding (Figure 3B). No viral RNA was identified in fecal and nasal samples from control calves.

### Gross lesions

**Figure 4:**
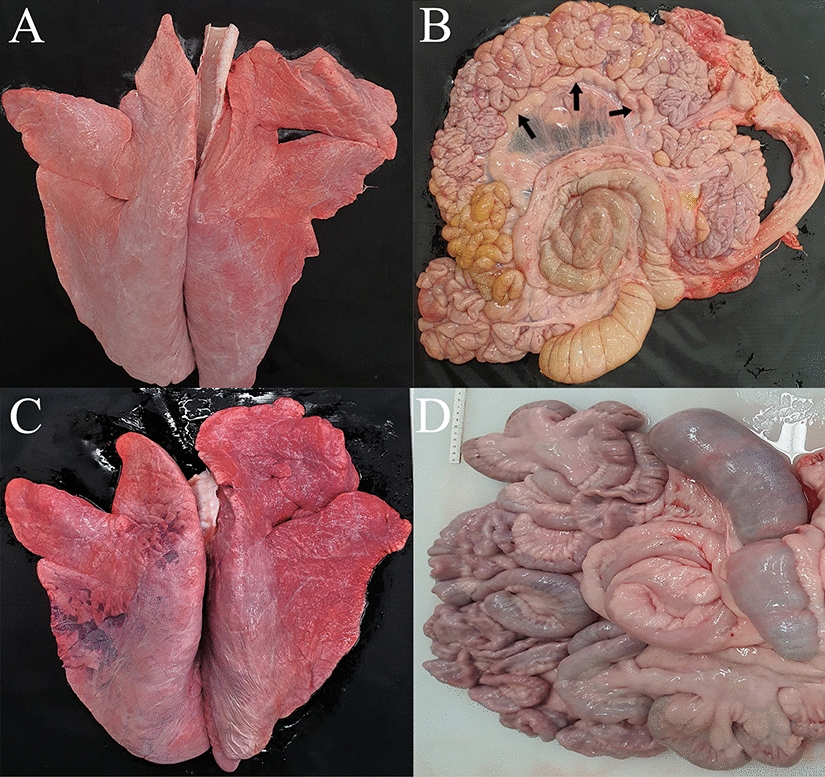
**Gross lesions in BCoV-infected and control calves.** Infected calves showed no macroscopic lesions in the lungs (**A**) or intestines (**B**) but had enlarged mesenteric lymph nodes (**B**, arrow). Control calves lacked detectable lesions in the lungs (**C**) and intestines (**D**).

Necropsy of BCoV-infected calves revealed no characteristic gross lesions in the lung (Figure 4A), small or large intestines (Figure 4B), or other organs. However, MLNs were consistently enlarged in all infected calves (Figure 4B). Control calves showed no gross changes (Figures 4C and D).

### BCoV detection in tissues

The presence of BCoV in different tissues was evaluated by RT-dPCR (Table 3). The assay detected the N gene over a dynamic range of 0–13 960 copies/μL. Viral RNA was present in the liver (3–3760 copies/μL), kidneys (1–3 copies/μL), tonsils (0.05–61 copies/μL), respiratory tract (2–4 copies/μL), and digestive tract (42–13 960 copies/μL) of every infected calf. In contrast, the spleen (0–2 copies/μL) and MLN (0.2–2 copies/μL) were positive in only two calves. No viral RNA was detected in any tissue from control calves.

**Table 3:** **Histopathological lesions and BCoV antigen detection in tissue samples from four BCoV-infected Holstein calves assessed by RT-dPCR and IHC**

Tissue	Histopathological lesions								RT-dPCR								IHC							
	# 1	# 2	# 3	# 4	# 5	# 6	# 7	# 8	# 1	# 2	# 3	# 4	# 5	# 6	# 7	# 8	# 1	# 2	# 3	# 4	# 5	# 6	# 7	# 8
Tonsil	1	1	1	0	N/A	N/A	N/A	N/A	+	+	+	–	N/A	N/A	N/A	N/A	+	+	+	–	N/A	N/A	N/A	N/A
Trachea	0	1	1	0	0	N/A	1	N/A	+	+	+	+	+	N/A	+	N/A	–	+	+	+	–	N/A	+	N/A
Lung	0	0	1	1	0	0	1	1	+	+	+	+	+	+	+	+	+	+	+	+	+	+	+	–
Liver	0	1	0	1	0	0	0	0	+	+	+	+	+	+	+	+	+	+	+	+	+	+	+	+
Kidney	0	0	0	0	0	0	0	0	+	+	+	+	+	+	+	+	+	+	+	+	+	+	+	+
Spleen	0	0	0	0	0	0	0	0	–	+	+	–	–	+	+	–	–	–	–	–	–	–	–	–
MLN	0	0	0	0	0	0	0	N/A	+	+	–	–	+	+	+	N/A	–	–	–	–	–	–	–	N/A
Abomasum	0	0	0	1	0	0	0	1	+	+	+	+	+	+	+	+	+	+	+	+	+	+	+	+
Small Int	1	1	1	1	0	1	0	1	+	+	+	+	+	+	+	+	+	+	+	+	+	+	+	+
Large Int	1	1	1	1	1	0	0	1	+	+	+	+	+	+	+	+	+	+	+	+	+	+	+	+

BCoV: bovine coronavirus, RT-dPCR: digital reverse-transcription polymerase chain reaction, IHC: immunohistochemistry, MLN: mesenteric lymph node, Small Int.: small intestine, Large Int.: large intestine.

Histopathology score: 0: normal; 1: lesion present.

N/A: not applicable (because these samples were not collected).

–: negative; +: positive.

### Histological evaluation

**Figure 5:**
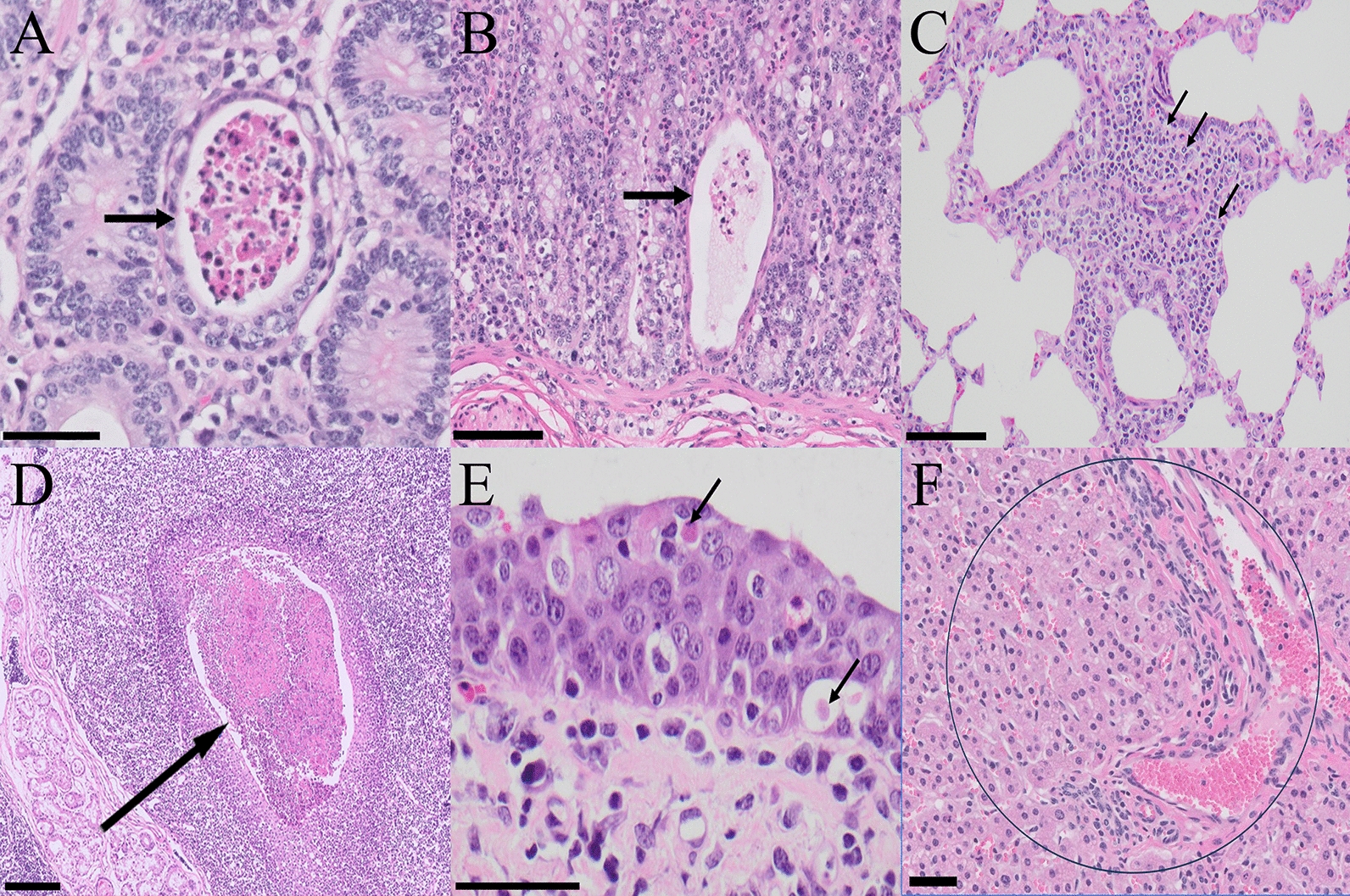
**Histopathological changes in BCoV-infected calves.** Infected calves showed necrotizing enteritis in the small intestine (**A**), crypt dilatation with necrotic debris and neutrophils in the abomasum (**B**), mild peribronchiolar and perivascular lymphohistiocytic infiltration in the lung (**C**), necrotic material and degenerated neutrophils in the tonsillar crypt (**D**), necrosis of the mucosal epithelium and infiltration of mononuclear inflammatory cells in the submucosa in the trachea (**E**), and lobular hepatitis with apoptotic and ballooned hepatocytes (**F**). Arrows (**A–E**) and circle (**F**) mark lesions. Scale bars: **A**, **B** and **E** = 100 μm; **C** and **F** = 50 μm; **D** = 200 μm.

Histological changes did not mirror the gross findings. In BCoV-infected calves, mild-to-moderate lesions were observed in the small and large intestines. Lungs were largely unremarkable, with occasional mild histopathological lesions (Table 3). The small intestine showed villous atrophy, surface enterocyte exfoliation, and necrotizing enteritis (Figure 5A). Multifocal cryptitis with necrotic neutrophils and cellular debris affected the large-intestinal mucosa. The abomasum exhibited lamina propria infiltration and mild cryptitis containing necrotic inflammatory cells and debris (Figure 5B). Two calves had mild peribronchiolar and perivascular infiltration of mononuclear infiltrates in the lungs (Figure 5C). The tonsillar crypts contained multifocal accumulation of necrotic debris and degenerated neutrophils in three calves (Figure 5D). In two calves, the tracheal mucosa displayed epithelial necrosis with submucosa mononuclear infiltrates (Figure 5E). In addition, in the liver, the hepatic parenchyma exhibited multifocal, mixed inflammatory infiltrates with central necrosis; a necrotic foci contained degenerated neutrophils and inflammatory cells (Figure 5F). The kidneys, MLN, and spleen were histologically normal in all animals. Collectively, enteritis, mild interstitial pneumonia, hepatitis, and mild tracheal inflammation were notable histopathological features.

### BCoV detection using IHC

**Figure 6:**
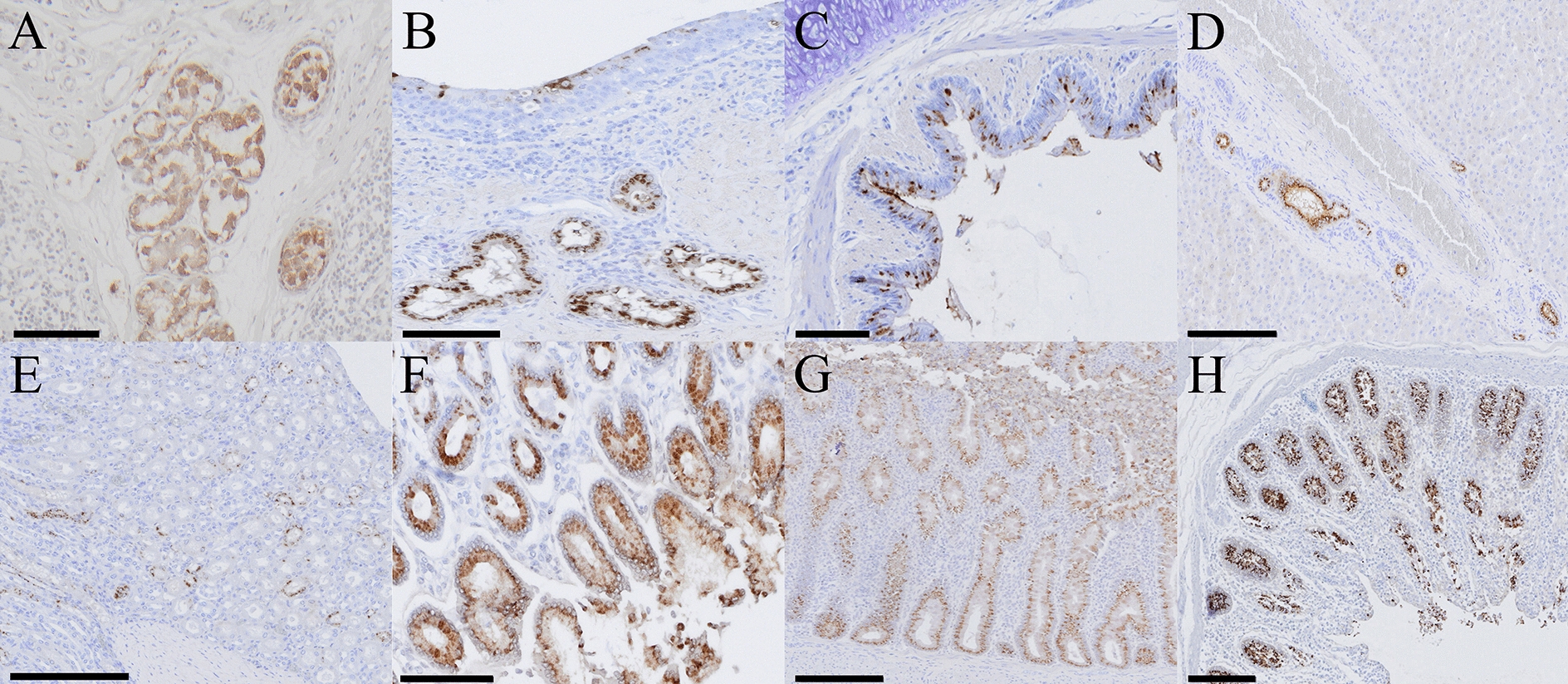
**IHC detection of BCoV antigens in Holstein calves inoculated intranasally with an enteric BCoV isolate.** Positive immunostaining (brown) was observed in tonsillar epithelial cells (**A**), tracheal glands (**B**), bronchiolar epithelium (**C**), biliary duct epithelial cells in the liver (**D**), renal tubular epithelial cells (**E**), the abomasal glandular epithelium (**F**), and crypt epithelial cells of the ileum (**G**) and colon (**H**). Scale bars: panels **A**–**C** and **F** = 100 μm; panels **D**, **E**, **G**, and **H** = 200 μm.

BCoV antigen distribution was evaluated by IHC in tissues collected from infected calves; results are summarized in Table 3. Antigen-positive cells were identified in tonsillar epithelium (Figure 6A), tracheal glands (Figure 6B), bronchiolar epithelium (Figure 6C), biliary duct epithelial cells (Figure 6D), renal tubular epithelium (Figure 6E), and in the crypt epithelial cells of the abomasum (Figure 6F), ileum (Figure 6G), and colon (Figure 6H). BCoV antigen was additionally identified within lymphocytes of Peyer’s patches. The respiratory tract contained fewer antigen-positive cells than the digestive tract. Within the gut, more immunoreactive cells were observed in the large intestine than in the small intestine, showing no concordance between BCoV antigen distribution and macroscopic lesions. This report is the first to demonstrate BCoV antigens in the abomasum, liver, and kidney tissue. Despite RT-dPCR positivity, IHC failed to reveal viral antigens in the spleen or MLN. Findings from sham-inoculated controls are provided in Additional file 1.

## Discussion

Coronaviruses (CoVs) cause respiratory, enteric, neurologic, and hepatic diseases in many animal species [4, 47–51]. BCoV is a major contributor to calf diarrhea, yet its respiratory pathogenicity remains contested despite dual mucosal tropism. Here, we characterized clinical signs, virus excretion, lesions, and tissue distribution after intranasal inoculation of Holstein calves with a fecal BCoV isolate. Using RT-dPCR and IHC, we demonstrate for the first time, viral dissemination to the liver and kidneys beyond respiratory and digestive tracts, underscoring its extensive tissue tropism.

Phylogenetic analysis based on the S gene showed that the fecal BCoV isolate clustered with recently identified Korean enteric BCoVs. The isolate exhibited 97.0–97.2% identity with respiratory BCoVs (OK and LSU strains) reported in the USA. Furthermore, comparison of genetic differences between respiratory and enteric BCoVs revealed no distinctive signatures, likely reflecting limited genetic information available for respiratory strains. These findings imply that BCoVs circulating worldwide appear to have no clear boundary between respiratory and enteric tropism. Consequently, variations in the S gene potentially may contribute to uncertainty in tissue tropism between respiratory and enteric BCoVs, suggesting overlap in tissue tropism at the pathological level. Future studies should prioritize additional surveillance and genetic characterization of respiratory isolates.

Infected calves developed moderate-to-severe diarrhea without nasal discharge or cough. This contrasts with our previous study, in which nasal discharge was the common symptom [34]. The big discrepancy between the two studies was the age of calves, suggesting that younger calves likely exhibit heightened susceptibility to enteric infection. Consistent with earlier work in 5-day-old calves [7, 52], diarrhea predominates when infection occurs during the first 2 weeks of life [1]. Disease severity seems strongly age-dependent. Although viral shedding occurs nasally, overt respiratory disease typically arises between 2 and 6 months of age [3]. Experimental inoculations rarely reproduce respiratory symptoms, possibly because they are mild or require additional stressors such as coinfection, immunosuppression, or management factors [7, 11, 37, 48]. Nevertheless, the virus’s potential to contribute to respiratory disease in the field should not be underestimated.

Our data show that nasal shedding began 2 days before fecal shedding in every calf. This pattern agrees with earlier reports in BCoV-infected Holsteins, where nasal excretion also preceded fecal shedding [8, 34]. No calf developed respiratory signs despite continuous nasal shedding, indicating that the presence of BCoV RNA in nasal swabs does not necessarily predict respiratory disease. BCoV shedding from both nasal and fecal swabs persisted until the study end point in every animal, echoing previous observations of concurrent secretion at the two sites [11, 33, 48]. We, therefore, infer that BCoV replicates in the respiratory tract for several days after initial exposure. Diarrhea appeared 2 days after fecal shedding, implicating fecal virus excretion in enteric disease; this interval differs from that reported in our previous work [34]. Whether the lack of respiratory illness reflects the enteric origin of the inoculum, calf age, or vaccine-mediated protection remains unresolved at this time. Collectively, our findings suggest that early replication in the respiratory tract may facilitate subsequent viral dissemination after intestinal infection. The calf that developed diarrhea at 1 dpi shed BCoV in feces from 3 to 9 dpi; another calf that first developed diarrhea at 3 dpi began fecal shedding at 5 dpi and continued through 9 dpi.

Previous studies describe macroscopic lesions in BCoV-infected cattle that include fluid-filled intestines, abomasal distention, catarrhal alterations in the distal ileum, spiral colon with mucosal and serosal congestion, cardiac petechiae, centrilobular hepatic necrosis, and MLN enlargement [19, 20, 52, 53]. Reported pulmonary changes comprise mild lesions, congestion, and pneumonic foci [1, 9, 19, 54]. Aside from MLN enlargement, no gross changes were evident in most tissues of the infected Holstein calves in the present study. Notably, despite universal diarrhea, the small and large intestines lacked visible lesions, diverging from earlier descriptions [1, 7, 36, 53, 55]. Similarly, the lungs, liver, and kidneys appeared normal. Nevertheless, we detected BCoV not only in the respiratory and digestive tracts but also in the liver and kidneys as determined by RT-dPCR, even in the absence of lesions. In contrast, the spleen appears unsuitable for detection, as viral RNA was found by RT-dPCR in only four calves and was undetectable by IHC. Taken together, although an enteric BCoV isolate was used, the virus may not be limited to intestinal lesions.

Enteritis, tonsillitis, mild interstitial pneumonia, tracheitis, and hepatitis developed in calves inoculated with BCoV. Histological lesions were generally mild-to-moderate and occurred more often in the small intestine than in the large intestine. Consistent with earlier work, we noted progressive villous atrophy in the small intestine [7]; however, we did not observe the colonic crypt hyperplasia and ridge atrophy by Clark [1]. Such discrepancies likely reflect differences in inoculation route, viral dose, calf age, and colostrum intake. The comparatively limited digestive lesions in the present study may stem from our lower challenge dose and protective effect of colostrum [7, 56]. Previous studies also documented tracheal and pulmonary epithelial injury in infected calves [7], findings reported in several of our animals. Although tonsillar inflammation was not universal, its presence indicates that this organ merits routine examination during BCoV infection. Our data, therefore, support the intranasal inoculation as a practical model for BCoV infection, even though some differences from oral challenge remain.

The present work is the first to demonstrate BCoV-associated hepatitis. Other CoVs, including SARS-CoV-2, SARS-CoV, and murine hepatitis virus (MHV), also provoke hepatic inflammation [57–61]. The histopathologic lesions characterized by necrotic foci and inflammatory infiltrates with neutrophils and mononuclear cells are consistent with liver pathology induced by MHV [62]. Liver dysfunction occurs in 14–53% of patients with coronavirus disease-19 (COVID-19) [63–67], yet the mechanisms underlying SARS-CoV-2 induced liver injury remain poorly defined. Proposed mechanisms include direct cytopathic effects and dysregulated immune responses that amplify inflammatory signaling [68]. SARS-CoV and SARS-CoV-2 use the angiotensin-converting enzyme 2 receptor for cellular entry, which is upregulated in biliary epithelial cells of infected patients. This pattern suggests that these viruses may directly infect bile ducts, leading to liver dysfunction [69]. At present, the route by which BCoV infects the liver cannot be defined; however, the results indicate that BCoV may cause liver damage [20]. Although BCoV antigen was detected only in biliary duct epithelial cells and not in hepatocytes, infected calves exhibited lobular hepatitis with apoptotic and ballooned hepatocytes and focal necrosis, consistent with histological lesions reported in other CoVs [62, 69–71]. To date, evidence for BCoV-associated hepatitis remains limited, and the pathogenic mechanisms underlying hepatic involvement are not yet fully understood. Whether BCoV-related hepatitis has simply been overlooked, never occurred, or reflects newly acquired tissue tropism is uncertain. Our findings nevertheless indicate that, similar to MHV, BCoV is polytropic, and further investigation into its hepatic pathogenicity is warranted.

IHC demonstrated BCoV antigens in epithelial cells of the tonsils, trachea, lungs, liver, kidneys, abomasum, and both small and large intestines. In contrast, viral antigens were absent from the spleen and MLNs despite PCR positivity. Immune-cell-rich tissues such as the spleen and MLN may be less supportive of active replication than epithelia. To our knowledge, this is the first report of BCoV antigens in the abomasal glandular epithelium, biliary epithelium, and renal tubules. Moreover, antigen loads were consistently higher in the gastrointestinal tract than in the respiratory tract, likely reflecting the isolate’s enteric origin. Thus, the enteric isolate used here can disseminate to multiple organs, even when respiratory, hepatic, or renal lesions are unapparent. A similar disconnect between viral presence and gross pathology is well recognized for infectious bronchitis virus in chicken [72, 73]. In our calves, large-intestinal antigen loads exceeded those in the respiratory tract despite the absence of macroscopic colonic lesions, aligning with previous observations [7], supporting the concept of sequential respiratory-to-enteric replication [49], perhaps indicating viral persistence in a latent or subclinical state [74, 75]. Collectively, these data expand the recognized tissue range of BCoV and underscore its capacity to produce multifocal, often subclinical, infection.

The main limitation of this study was the use of antibody-positive calves. In the ROK, mother cows are routinely vaccinated against BCoV and BoRVA, and thus most calves have maternal antibodies. In addition, the viral dose used here was lower than in other studies [7, 22, 75–77], highlighting the absence of established dose for experimental BCoV infection. Nevertheless, BCoV infection was established in colostrum-fed Holstein calves, causing diarrhea. Although the study has weaknesses that constrain conclusions, a novel finding is the varied tissue tropisms following infection with an enteric BCoV isolate.

## Conclusions

BCoV initially replicates in respiratory epithelial cells, irrespective of subsequent gastrointestinal involvement or clinical status. Despite the enteric origin of the isolate, BCoV-infected Holstein calves developed enteritis, tonsillitis, mild interstitial pneumonia, and hepatitis. IHC identified viral antigens in epithelial cells of the tonsils, trachea, lungs, liver, kidneys, abomasum, and both small and large intestines; antigen presence did not correlate with gross or microscopic lesions. Viral antigen was more abundant in the digestive than in the respiratory tract. Detection of viral antigen in the liver and kidneys, in addition to the respiratory and gastrointestinal tracts, underscores the broad tissue tropism of this enteric BCoV isolate and its capacity to induce extra-respiratory and extra-intestinal disease. Collectively, these findings extend current understanding of BCoV pathogenesis.

## Supplementary Information

**Additional file 1. IHC results of tissues from negative-control calves.** No viral antigens were detected in the tonsil, tracheal glands, bronchioles, liver, kidneys, abomasum, or smalland large intestines. Scale bars: panels A, E, F, and G = 100 μm; panels B, C, D, and H = 200 μm.

## Data Availability

No datasets were generated or analyzed during the current study. All data supporting the findings of this study are included in the manuscript and its additional file.
