# Supplementary figures and images for: Experimental intranasal infection reveals broad tissue tropism of bovine coronavirus
Source: Vet Res. 2026 Jan 3;57:27. doi: 10.1186/s13567-025-01703-9 (PMC12866565; doi:10.1186/s13567-025-01703-9)

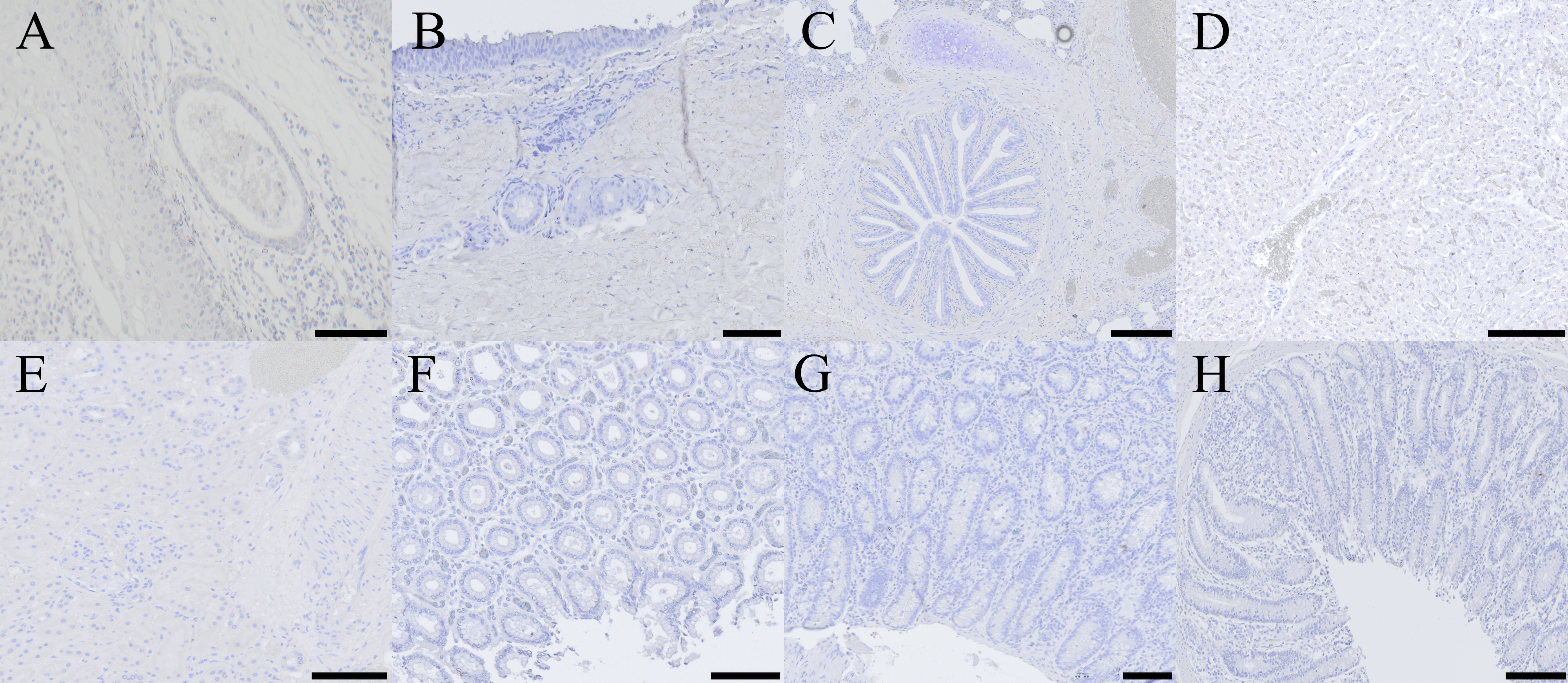

Supplement: Supplementary file 1 — Additional file 1. IHC results of tissues from negative-control calves. No viral antigens were detected in the tonsil, tracheal glands, bronchioles, liver, kidneys, abomasum, or smalland large intestines. Scale bars: panels A, E, F, and G = 100 μm; panels B, C, D, and H = 200 μm. [file 13567_2025_1703_MOESM1_ESM.tif]
